# Estrogen receptor variants in ER-positive basal-type breast cancers responding to therapy like ER-negative breast cancers

**DOI:** 10.1038/s41523-019-0109-7

**Published:** 2019-04-18

**Authors:** Floris H. Groenendijk, Tina Treece, Erin Yoder, Paul Baron, Peter Beitsch, William Audeh, Winand N. M. Dinjens, Rene Bernards, Pat Whitworth

**Affiliations:** 1 Department of Pathology, Erasmus MC Cancer Institute, Rotterdam, The Netherlands; 2 Agendia, Irvine, CA USA; 3 Breast and Melanoma Specialists of Charleston, Charleston, SC USA; 4 Dallas Surgical Group, Dallas, TX USA; 5 Division of Molecular Carcinogenesis, Oncode Institute, The Netherlands Cancer Institute, Amsterdam, The Netherlands; 6 Nashville Breast Center, Nashville, TN USA

**Keywords:** Breast cancer, Cancer genomics

## Abstract

Immunohistochemically ER-positive HER2-negative (ER+HER2−) breast cancers are classified clinically as Luminal-type. We showed previously that molecular subtyping using the 80-gene signature (80-GS) reclassified a subset of ER+HER2− tumors to molecular Basal-type. We report here that molecular reclassification is associated with expression of dominant-negative ER variants and evaluate response to neoadjuvant therapy and outcome in the prospective neoadjuvant NBRST study (NCT01479101). The 80-GS reclassified 91 of 694 (13.1%) immunohistochemically Luminal-type tumors to molecular Basal-type. Importantly, all 91 discordant tumors were classified as high-risk, whereas only 66.9% of ER+/Luminal-type tumors were classified at high-risk for disease recurrence (i.e., Luminal B) (*P* < 0.001). ER variant mRNA (ER∆3, ER∆7, and ERα-36) analysis performed on 84 ER+/Basal tumors and 48 ER+/Luminal B control tumors revealed that total ER mRNA was significantly lower in ER+/Basal tumors. The relative expression of ER∆7/total ER was significantly higher in ER+/Basal tumors compared to ER+/Luminal B tumors (*P* < 0.001). ER+/Basal patients had similar pathological complete response (pCR) rates following neoadjuvant chemotherapy as ER−/Basal patients (34.3 vs. 37.6%), and much higher than ER+/Luminal A or B patients (2.3 and 5.8%, respectively). Furthermore, 3-year distant metastasis-free interval (DMFI) for ER+/Basal patients was 65.8%, significantly lower than 96.3 and 88.9% for ER+/Luminal A and B patients, respectively, (log-rank *P* < 0.001). Significantly lower total ER mRNA and increased relative ER∆7 dominant-negative variant expression provides a rationale why ER+/Basal breast cancers are molecularly ER-negative. Identification of this substantial subset of patients is clinically relevant because of the higher pCR rate to neoadjuvant chemotherapy and correlation with clinical outcome.

## Introduction

Diagnostic testing of breast cancers for hormone receptor (HR) and HER2 status by immunohistochemistry and/or in-situ hybridization is routinely performed as an integral step to clinically define tumor characteristics and predict tumor behavior.^1,2^ Advancement of technology has made it possible to molecularly characterize these tumors at the genomic level by evaluating underlying and intrinsic differences in tumor biology.^3,4^ Although clinical subtypes overlap with these molecular subtypes, a significant number of patients will be reclassified based on the functionality of molecular pathways.^5,6^ This reclassification may have important consequences for treatment allocation, response and clinical outcome. For example, we have reported previously that HR+HER2− patients who are reclassified into Basal-type by the 80-gene signature (80-GS) have a substantially higher rate of pathologic complete response (pCR) to neoadjuvant chemotherapy compared to HR+HER2−/Luminal patients (32% vs. 5%).^7^ A contributor to molecular reclassification of estrogen receptor (ER) positive tumors might be ER borderline positivity by immunohistochemistry (IHC), i.e., 1–9% positive cells, since it has been shown that those tumors mainly cluster with Basal-type tumors by gene-expression profiling.^8,9^ However, molecular subtyping will also identify a group of clearly ER-positive patients (by IHC and/or mRNA) that fail to elicit the estrogen-induced transcriptional responses.

A possible explanation is the inability of IHC and/or mRNA to differentiate non-functional from functional ER in those patients. Several ERα variants have been described in human breast cancers and in normal tissues expressing ERα, most of them being mRNA exon skipping splicing variants.^10–12^ The activity of these variants has been investigated in multiple functional studies, i.e., they can be functionally negative, dominant negative or dominant active on ERα target genes.^10^ Two splice variants (ER∆3 and ER∆7) have been described as dominant negative forms in the presence of wild-type ERα, indicating that they also inhibit the function of wild-type ERα.^10,13^ More recently, a third variant with dominant negative activity has been reported, named ERα-36 (referring to the protein molecular weight of 36 kDa).^14^ We have shown previously in a small case-control study that ER+/Basal tumors have relatively high levels of the dominant-negative ER∆7 splice variant, although consequences for response to adjuvant therapy and clinical outcome were unknown in those cases.^15^

In the present study, we have examined the expression of total ER and ER variants mRNA in ER+/Basal breast cancer patients enrolled in the prospective neoadjuvant NBRST registry trial (NCT01479101) and compared these expression values to patients with ER+/Luminal B breast cancer. We also report pathological complete response rates to neoadjuvant therapy and 3-year follow-up data for both patient groups within the NBRST trial and compared those to ER−/Basal breast cancer patients.

## Results

### Clinical and molecular subtyping

A total of 1072 eligible patients were enrolled in the NBRST study, of which 694 (64.7%) patients were classified as ER-positive based on IHC (defined as ≥1% ER+ cells). The 80-gene molecular subtyping signature (BluePrint, Agendia) classified 514/694 (74.1%) ER-positive patients as Luminal-type, 91/694 (13.1%) ER-positive patients as Basal-type and 89/694 (12.8%) ER-positive patients as HER2-type, of which 86/89 (96.6%) were HER2-positive by IHC/FISH. MammaPrint, a 70-gene risk of recurrence signature, defined all 91 ER+/Basal patients at high-risk for disease recurrence. In contrast, the frequency of MammaPrint high-risk classification among 514 ER+/Luminal patients was 66.9% (i.e., Luminal B) (*P* < 0.001; Table 1).^16^ Both subgroups of ER+/Basal and ER+/Luminal B patients had a broad range of percentage positive staining for ER by IHC ranging from 1–99% for ER+/Basal and 1–100% for ER+/Luminal B patients, albeit a significant difference in the mean percentage positive ER staining was found (87.6% for ER+/Luminal B vs. 22.1% for ER+/Basal, *P* < 0.001; Table 1).^16^ The frequency of ER borderline tumors (1–9% positive cells by IHC) was 44.0% for ER+/Basal tumors and 1.2% for ER+/Luminal B tumors (*P* < 0.001; Table 1).^16^ The difference in ER staining was reflected in a significant difference in the percentage of PR-positive tumors between the two groups (85.2% for ER+/Luminal B vs. 29.7% for ER+/Basal, *P* < 0.001; Table 1) and the mean percentage PR positivity in PR-positive tumors (58.1% for ER+/Luminal B vs. 10.9% for ER+/Basal, *P* < 0.001; Table 1).^16^ This finding is consistent with PR expression being controlled transcriptionally, at least in part, by ER.

**Table 1:** ER/PR positivity and HER2 status in ER-positive tumors (IHC ≥1%) in NBRST (*N* = 694)

	80-GS Luminal A (*N* = 170)	80-GS Luminal B (*N* = 344)	80-GS Basal (*N* = 91)	80-GS HER2 (*N* = 89)
ER positivity				
Mean % staining	88.4%	87.6%	22.1%	60.0%
Range (min,max)	1,100%	1,100%	1,99%	1,100%
IQR^a^ (Q1-Q3)	90–99%	85–99%	4–29%	20–90%
% ER borderline^b^	2.9%	1.2%	44.0%	15.7%
PR status				
Positive (≥1% IHC)	96.5%	86.6%	29.7%	62.9%
Mean % staining^c^	76.8%	58.1%	10.9%	31.6%
Range (min,max)	1,100%	1,100%	1,50%	1,100%
IQR^a^ (Q1-Q3)	70–98%	28–90%	2–9%	5–51%
% HER2 positive (IHC/FISH)	10.6%	19.5%	16.5%	96.6%

^a^IQR, interquartile range

^b^defined as 1–9% positive cells by IHC

^c^mean % staining in PR-positive tumors

### Total ERα and ERα variant mRNA qPCR analysis

**Fig. 1:**
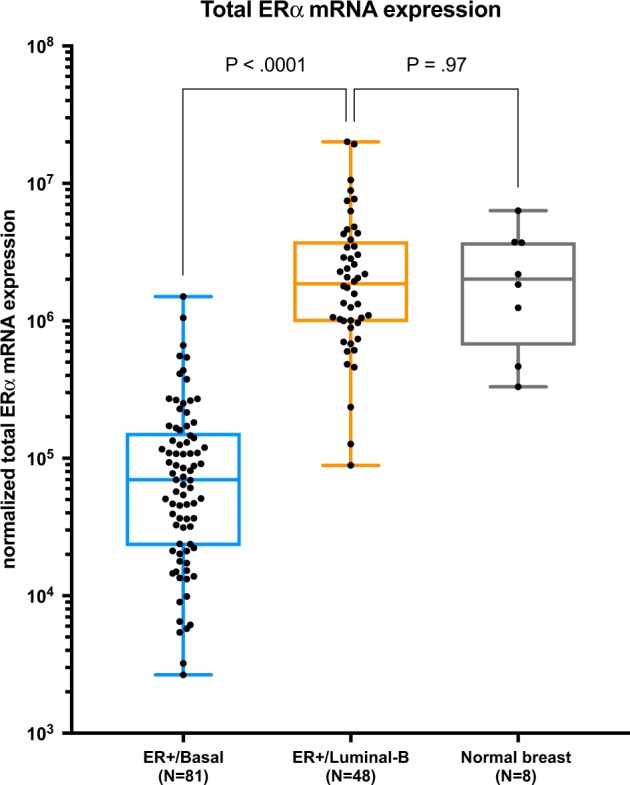
Normalized total ERalpha mRNA expression. Box plots showing normalized total ERalpha mRNA expression for ER+/Basal tumors (*N* = 81, blue box), ER+/Luminal B tumors (*N* = 48, orange box) and normal breast tissues (*N* = 8, gray box). Central line in boxes represent median value, boundaries of boxes represent the interquartile range and ends of whiskers represent the minimum and maximum values. *P*-values are obtained using the Mann–Whitney test

Total ERα mRNA expression measured by qPCR was significantly lower for ER+/Basal tumors compared to a control group of ER+/Luminal B tumors (*P* < 0.001; Fig. 1).^16^ Interestingly, the levels of total ERα mRNA for ER+/Luminal B tumors were similar to total ERα mRNA expression in the control group of normal breast tissues (*P* = 0.97; Fig. 1).^16^

**Fig. 2:**
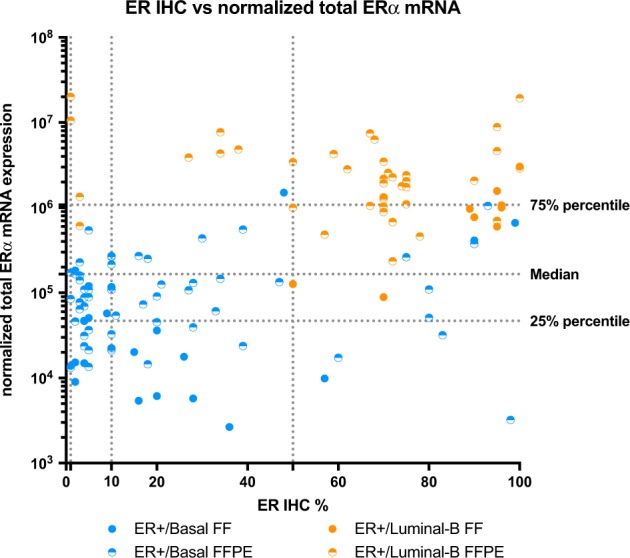
Correlation between percentage ER staining by immunohistochemistry and normalized total ERalpha mRNA expression. Distribution plot showing the correlation between percentage ER staining by immunohistochemistry and normalized total ERalpha mRNA expression for ER+/Basal tumors (*N* = 81, blue dots) and ER+/Luminal B tumors (*N* = 48, orange dots). FF fresh frozen samples; FFPE formalin-fixed paraffin embedded samples

Figure 2 illustrates the relationship between total ERα mRNA expression and the percentage of ER positivity by IHC.^16^ Interestingly, most ER+/Basal tumors showed relatively low total ERα mRNA expression, even in cases of high percentage positive staining by IHC. In contrast, ER+/Luminal B tumors showed relatively high total ERα mRNA expression, even in borderline (defined as ER 1–9% by IHC) or low (defined as ER <50% by IHC) ER expressing tumors (Fig. 2).^16^

**Fig. 3:**
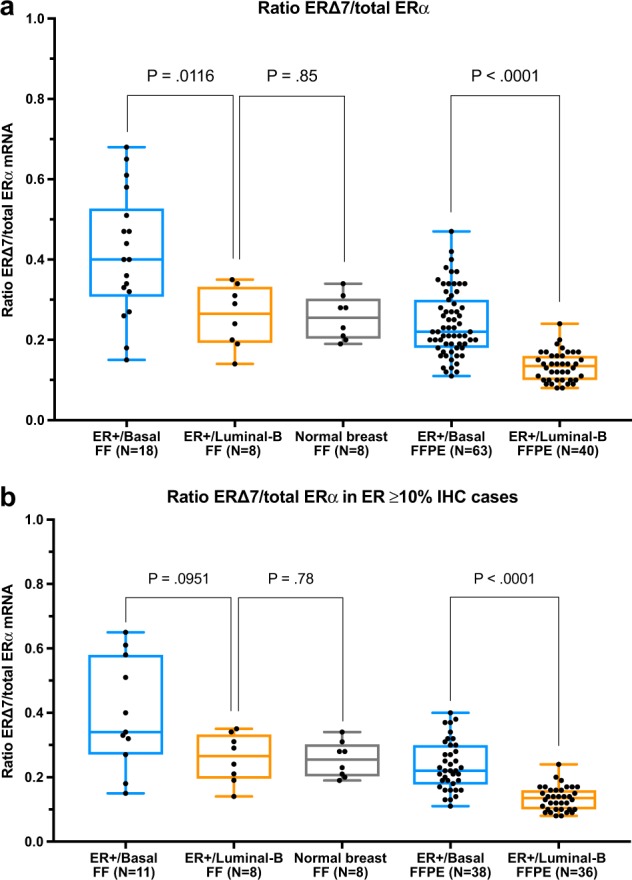
Ratio of ER∆7 mRNA expression and total ERalpha mRNA expression. Box plots showing ratio of ER∆7 mRNA expression and total ERalpha mRNA expression for ER+/Basal tumors (blue boxes), ER+/Luminal B tumors (orange boxes) and normal breast tissues (gray box). Ratio’s for fresh frozen (FF) samples and formalin-fixed paraffin embedded (FFPE) samples are depicted in separate boxplots. Central line in boxes represent the median value, boundaries of boxes represent the interquartile range and ends of whiskers represent the minimum and maximum values. *P*-values are obtained using the Mann-Whitney test. **a** Box plots for all ER+/Basal and ER+/Luminal B tumors. **b** Box plots for subgroup of ER+/Basal and ER+/Luminal B tumors with ER-positivity of ≥10% by immunohistochemistry

Expression of dominant negative ERα variants (ER∆3, ER∆7, and ERα-36) was measured by variant-specific quantitative real-time PCR. It should be noted that the ERα-36 variant could only be reliably measured in cDNA from fresh frozen RNA samples because of the low mRNA expression of this variant, which was below the detection limit in transcribed cDNA from most FFPE RNA samples. As shown in Supplemental Fig. 1, the ER∆7 variant was the highest expressed variant in both ER+/Basal tumors (Fig. S1A-B), as well as ER+/Luminal B (Fig. S1C-D) and normal breast (Fig. S1E).^16^ The absolute expression of ER∆7 was lower in ER+/Basal tumors compared to ER+/Luminal B tumors. However, the expression of ER∆7 relative to total ERα mRNA was significantly increased in ER+/Basal tumors compared to ER+/Luminal B tumors (*P* < 0.001; Fig. 3a).^16^ This difference in relative ER∆7 expression was present in the complete group of tumors with ER ≥1% by IHC (Fig. 1a), as well as in the subgroup of tumors with ER ≥10% by IHC (Fig. 3b).^16^ The expression of ER∆3 and ERα-36 relative to total ERα mRNA was much lower (ratio <0.05) than the relative ER∆7 expression and not significantly different between ER+/Basal and ER+/Luminal B tumors. The relative expression of ER∆3 is shown in Supplementary Fig. 2, for ERα-36 the relative expression was even lower and only measurable in fresh frozen samples (data not shown).^16^

### Treatment response and outcome

To compare treatment response and outcome across molecular subtypes, only HER2− patients with known ER status, 80-GS subtype, and follow-up were evaluated (*N* = 667). The majority of these HER2− patients were ER-positive by IHC (65.7%). The 80-GS and MammaPrint classified 130 ER-positive patients (29.7%) as Luminal A (low risk of recurrence), 238 ER-positive patients (54.3%) as Luminal B (high risk of recurrence) and 70 ER-positive patients (16.0%) as Basal-type. All 229 ER-negative patients were classified as Basal-type. The majority of these patients received neoadjuvant chemotherapy (610/667; 91.5%), whereas a small group of patients received neoadjuvant hormonal therapy (57/667; 8.5%). Following neoadjuvant treatment, 34.3% (24/70) of ER+/Basal patients had achieved a pathological complete response (pCR), similar to 37.6% (86/229) of ER−/Basal patients, and much higher than 5.9% (14/238) of ER+/Luminal B or 2.3% (3/130) of ER+/Luminal A patients. All patients with pCR received neoadjuvant chemotherapy. Subset analysis ER+/Basal patients showed a non-significant difference in pCR rate between PR-negative patients (*N* = 53, 39.6% pCR) and PR-positive patients (*N* = 17, 17.6% pCR) (*P* = 0.14).

**Fig. 4:**
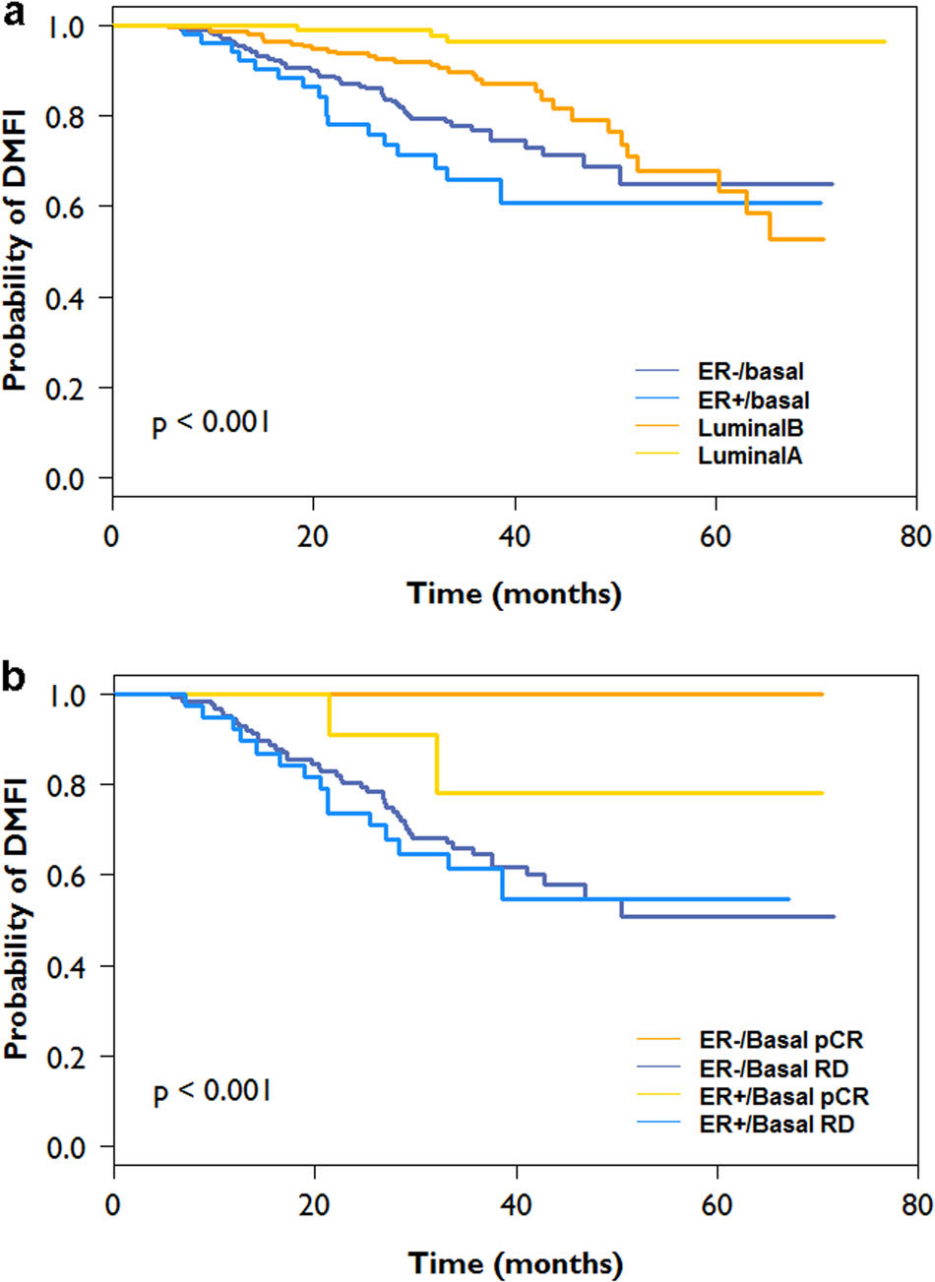
Kaplan Meier curves showing distant metastasis free interval (DMFI). Kaplan Meier curves showing distant metastasis free interval (DMFI) for HER2-negative patients in NBRST study with follow-up (*N* = 538). *P*-values are obtained using the log-rank test. **a** DMFI for all patients stratified by ER status and molecular subtyping. **b** DMFI of Basal patients (*N* = 252) stratified by ER status and response to neoadjuvant therapy (pCR pathological complete response or RD residual disease)

To evaluate long-term response, 583 patients from this subset reported follow-up with a median of 34 months (range 0.3–77 months). Clinical characteristics and outcome are shown in Table 2.^16^ The 3-year distant metastasis free interval (DMFI) for ER+/Basal patients was 65.8% (95%CI: 53.3–81.3), compared to 76.7% (95%CI: 70.5–83.5) for ER−/Basal patients (Table 2; Fig. 4a).^16^ ER+/Luminal patients had a substantially better 3-year DMFI: 88.9% (95%CI: 84.2–93.8) for ER+/Luminal B and 96.3% (95%CI: 92.3–100) for ER+/Luminal A patients (Table 2; Fig. 4a).^16^ A large proportion of Basal-type patients had a DMFI event in 3-years: 18 events in 54 ER+/Basal patients (33.3%) and 46 events in 198 ER−/Basal patients (23.2%). In contrast, there were 35 DMFI events in 213 ER+/Luminal B patients (16.4%) and 4 events in 118 ER+/Luminal A patients (3.4%). Of interest, all events in the ER+/Basal patients occurred before 4-years and mirrors the recurrence profile of ER−/Basal tumors as compared to Luminal B tumors where events are known to occur in years 5–10. Overall, there was a significant difference in DMFI survival between the ER+/Basal, ER−/Basal, ER+/Luminal B and ER+/Luminal A patients (log-rank *P* < 0.001; Fig. 4a).^16^ Subgroup analysis for ER+/Basal patients as compared to ER+/Luminal B patients showed a significant difference in DMFI survival (*P* = .002).

**Table 2:** Clinical characteristics of HER2-negative patients in NBRST study with follow-up (*N* = 538)

	All	ER+/Luminal A	ER+/Luminal B	ER+/Basal	ER−/Basal
	(*N* = 583)	(*N* = 118)	(*N* = 213)	(*N* = 54)	(*N* = 198)
Age					
<35	40 (6.9%)	3 (2.5%)	14 (6.6%)	8 (14.8%)	15 (7.6%)
35–50	208 (35.7%)	41 (34.7%)	68 (31.9%)	20 (37.0%)	79 (39.9)
51–70	287 (59.2%)	56 (47.5%)	111 (52.1%)	22 (40.7%)	98 (49.5%)
>70	48 (8.2%)	18 (15.3%)	20 (9.4%)	4 (7.4%)	6 (3.0%)
Ethnicity					
African/black	82 (14.1%)	8 (6.8%)	32 (15.0%)	5 (9.3%)	37 (18.7%)
Asian	12 (2.1%)	2 (1.7%)	7 (3.3%)	2 (3.7%)	1 (0.5%)
Caucasion/white	428 (73.4%)	101 (85.6%)	149 (70.0%)	42 (77.8%)	136 (68.7%)
Hispanic	52 (8.9%)	6 (5.1%)	22 (10.3%)	3 (5.6%)	21 (10.6%)
Mixed	4 (0.7%)	1 (0.8%)	–	1 (1.9%)	2 (1.0%)
Native America	2 (0.4%)	–	2 (0.9%)	–	–
Other	3 (0.5%)	–	1 (0.5%)	1 (1.9%)	1 (1.0%)
Menopausal status					
Post-menopausal	330 (56.6%)	74 (62.7%)	125 (58.7%)	24 (44.4%)	107 (54.0%)
Premenopausal and perimenopausal	247 (42.4%)	43 (36.4%)	88 (41.3%)	30 (55.6%)	86 (43.4%)
Unknown	6 (1.1%)	1 (0.8%)	–	–	5 (2.5%)
cT stage					
cT1	81 (13.9%)	14 (11.9%)	23 (10.8%)	6 (11.1%)	38 (19.2%)
cT2	331 (56.8%)	66 (55.9%)	119 (55.9%)	33 (61.1%)	113 (57.1%)
cT3	139 (23.7%)	34 (28.8%)	58 (27.2%)	10 (18.5%)	37 (18.7%)
cT4	31 (5.3%)	4 (3.4%)	12 (5.6%)	5 (9.3%)	10 (5.1%)
cTx	1 (0.2%)	–	1 (0.5%)	–	–
cN stage					
cN0	230 (39.5%)	59 (50.0%)	61 (28.6%)	22 (40.7%)	88 (44.4%)
cN1	268 (46.0%)	42 (35.6%)	117 (54.9%)	23 (42.6%)	86 (43.4%)
cN2	41 (7.0%)	6 (5.1%)	21 (9.9%)	4 (7.4%)	10 (5.1%)
cN3	14 (2.4%)	2 (1.7%)	3 (1.4%)	3 (5.6%)	6 (3.0%)
cNx	30 (5.1%)	9 (7.6%)	11 (5.2%)	2 (3.7%)	8 (4.0%)
Histological subtype					
Invasive carcinoma NST	489 (83.9%)	80 (67.8%)	170 (79.8%)	53 (98.1%)	186 (93.9%)
Invasive lobular carcinoma	63 (10.8%)	31 (26.3%)	25 (11.7%)	1 (1.9%)	6 (3.1%)
Mixed subtype	17 (2.9%)	4 (3.4%)	11 (5.2%)	–	2 (1.0%)
Other	14 (2.4%)	3 (2.5%)	7 (3.3%)	–	4 (2.0%)
Histological grade					
Grade 1	51 (8.7%)	31 (26.3%)	16 (7.5%)	1 (1.9%)	3 (1.5%)
Grade 2	193 (33.1%)	63 (53.4%)	96 (45.1%)	5 (9.3%)	29 (14.6%)
Grade 3	316 (54.2%)	16 (13.6%)	87 (40.8%)	48 (88.9%)	165 (83.3%)
N/A	23 (3.9%)	8 (6.8%)	14 (6.6%)	–	1 (0.5%)
MammaPrint genomic risk					
Low-risk	118 (20.2%)	118 (100%)	–	–	–
High-risk	465 (79.7%)	–	213 (100%)	54 (100%)	198 (100%)
Neoadjuvant therapy					
Chemotherapy	525 (90.1%)	80 (67.8%)	195 (91.5%)	53 (98.1%)	197 (99.5%)
Endocrine therapy	49 (9.3%)	34 (28.8%)	15 (7.0%)	–	–
N/A	9 (1.5%)	4 (3.4%)	3 (1.4%)	1 (1.9%)	1 (0.5%)
Adjuvant therapy					
Endocrine therapy	329 (56.4%)	102 (86.4%)	180 (84.5%)	32 (59.3%)	15 (7.6%)
None	254 (43.6%)	16 (13.6%)	33 (15.5%)	22 (40.7%)	183 (92.4%)
% DMFI at 3-years		96.3%	88.9%	65.8%	76.7%
(95% CI)		(92.3–100)	(81.2–93.8)	(53.3–81.3)	(70.5–83.5)

Subset analysis of ER+/Basal patients showed a non-significant difference in DMFI between PR-negative patients and PR-positive patients (Supplementary Fig. 3A, log-rank P = 0.08).^16^ Of note, PR-positive patients had worse DMFI than PR-negative patients in this subset as compared to the overall cohort where PR-positive patients are commonly Luminal-type and have a better DMFI than PR-negative patients (Supplementary Fig. 3B, log-rank *P* = 0.03).^16^ This apparent contradiction can be explained by the higher pCR rate for PR-negative patients in the ER+/Basal cohort. Basal-type patients who achieved a pCR exhibited significantly better 3-year probability of DMFI compared to those with residual disease (RD), regardless of ER status (log-rank *P* < 0.001; Fig. 4b).^16^ The 3-year DMFI for ER+/Basal patients with pCR was 78% (95% CI: 54.6–100%) compared to 61% (95% CI: 47.1–79.7%) for ER+/Basal patients with RD.

## Discussion

The question of whether a breast cancer is ER-positive and endocrine responsive has been an ongoing debate since the outset of endocrine therapy. Discordant ER status by immunohistochemistry and molecular subtype may arise from borderline ER expression (degree of positivity)^8^, testing artefacts resulting in inaccurate ER status determination^6,17^ or it may indicate the expression of a non-functional estrogen receptor. Gene signatures may help to resolve these uncertainties and more reliably and robustly classify patients based on activity within the molecular pathways. In the end biology matters, and it is more than just expression of ER.

To explain discordant clinical and molecular subtyping, we assessed 80-GS molecular subtype, total ER mRNA expression and ER variant mRNA expression in 84 ER+/Basal and 48 ER+/Luminal B breast cancers in a prospective trial (NBRST registry trial). We did not include ER−/Basal breast cancers for this analysis, since ER mRNA expression in those tumors is usually below the limit of detection. We asked whether molecular reclassification was associated with the level of total ER mRNA and expression of dominant-negative estrogen receptor variants. ER-positive patients, reclassified by the 80-gene signature as molecularly Basal-type, had significantly lower total ER expression by IHC and mRNA compared to a control group of ER+/Luminal B patients. We showed that expression of the ER∆7 splice variant relative to total ER mRNA is significantly increased in ER+/Basal patients compared to ER+/Luminal B patients. This difference was present in both borderline ER-positive tumors (1–9% IHC) and ER-positive tumors with higher ER expression (≥10% IHC, up to 99%). This observation is in concordance with our previous report which was based on a much smaller and more heterogeneous retrospective cohort of ER+/Basal patients without clinical follow-up.^15^ The combination of low ER mRNA expression and high relative ER∆7 variant expression provides a rationale why these are classified as Basal-type, but would not have been identified as ER-IHC negative (as illustrated in Fig. 4 of ref. ^15^).^15^

Interestingly, the 80-GS used in this study to classify patients as molecular Luminal-type, Basal-type or HER2-type was developed using IHC status as a guide.^18^ Although supervised by standard HR and HER2 IHC status, the genes comprising each molecular subtype profile were identified as the most differentially regulated between clinical subtypes, offering a different level of granularity. Currently, no ‘gold standard’ for molecular subtyping of breast cancer has been defined and, although overlap exists, different gene sets are used in each classifier.^19^ Of the 80 genes in the 80-GS, 13 of 28 reporter genes used to classify tumors as Basal-type (46.4%) overlap with genes present in the intrinsic gene set reported by Perou et al.^4,18^ The 80-GS gene set used for molecular subtyping has only 4 genes in common with the MammaPrint gene set used for assessing risk of recurrence (2 genes in the Basal-subtype and 2 genes in the Luminal-subtype). Recently, it was reported within the I-SPY 2 trial that ER+/80-GS Basal-type tumors have higher expression of basal-type keratins (keratins 5/14/17) compared with ER+/Luminal-type tumors.^20^ This supports the finding of Cheang et al. who identified EGFR and cytokeratin 5/6 expression by immunohistochemistry as prognostic markers for basal-like breast cancers.^21^

ER∆7 was the highest expressed variant among those tested, which is in line with previous observations.^13^ We found very low mRNA expression values of the ER variant ERα-36, which has been described as a mediator of the ER non-genomic signaling pathway.^14^ This partly conflicts with a previous study reporting, based on ERα-36 protein expression using a custom antibody, high expression of this variant in a significant subset of ER-positive breast cancers.^22^

We also compared total ER mRNA expression of ER+/Basal and ER+/Luminal B tumors with an external control group of normal breast tissues from patients with concurrent breast cancer. We strikingly found that ER mRNA expression levels of ER+/Luminal B tumors are comparable to normal breast epithelium. However, the normal breast group is relatively small and unmatched. Future work studying ER mRNA expression in a larger cohort of matched normal tissues at the time of tumor detection is necessary to investigate the relationship between ER mRNA expression in normal breast and tumor subtypes.

This study has some limitations. First, clinical subtyping by IHC/FISH analysis was performed in local pathology laboratories according to their pre-existing protocols, which may have contributed to interinstitutional differences in assessment and classification. This, and the fact that the NBRST neoadjuvant trial is enriched for high-risk patients, might explain the higher frequency of molecular reclassification of ER + HER2− tumors in our study compared to what has been reported for adjuvant studies with centrally assessed IHC/FISH subtypes. The prospective, randomized phase III MINDACT study used centrally assessed IHC/FISH subtypes and found that 99/4718 (2.1%) ER-positive (IHC ≥1%) patients were reclassified as Basal-type by the 80-GS.^5^ This percentage is in line with what we have reported previously as discordancy rate between mRNA based ER classification and molecular subtyping.^15^ Iwamoto et al. reported that 29 out 465 (6.2%) ER-positive (IHC ≥1%) patients were classified as Basal-type by PAM50 and 17 (58.6%) of them had ER ≥10%.^8^ However, compared to central IHC testing, local assessment of ER status by IHC better reflects how testing for ER is performed in routine clinical practice. Furthermore, the reclassification frequency in NBRST is comparable to the recently reported reclassification frequency in the multicenter phase II neoadjuvant I-SPY 2 trial. In this trial, the 80-GS was applied to 375 ER+/HER2− patients and 29% of patients were classified as Basal-type.^20^ The frequency of molecular reclassification of ER IHC-positive cases to Basal-type by 80-GS was evaluated by site to assess if there was a site dependence. We found that 31 different sites contributed to this subset of patients, most with only 1 or 2 patients each. The 10 institutions who contributed more ER+/Basal patients also enrolled a higher proportion of patients to the study overall, indicating that there was not a bias in IHC quality. Some institutions used both local and national laboratories to process the IHC, and all institutions used one of the two commonly used monoclonal, commercially available antibodies (Ventana SP-1 and Leica 6F11). The Ventana SP-1 clone is raised against a synthetic peptide that corresponds to the C-terminal domain of the ER alpha molecule (Ventana antibody datasheet). This antibody will, beside wild-type ERalpha, most likely also recognize ER∆3 but not ER∆7 since this variant leads to a truncated ER protein.^15^ The Leica 6F11 clone is raised against recombinant estrogen receptor alpha protein (Leica antibody datasheet). It is unknown if this antibody will recognize any of the ER variants.

Second, immunohistochemical assessment of ER was performed in needle core biopsies taken before neoadjuvant treatment. Although the reported concordance rate with surgical excision specimens is high, but variable (80–99%), it may have contributed to false positive staining.^23^ It has been shown that cases with large tumor size (>2 cm) will more frequently have a change in biomarker after neoadjuvant chemotherapy as a result of tumor heterogeneity.^24^ The average tumor size in NBRST is much higher than for example the MINDACT study (71.6% T1, e.g., <2 cm, in MINDACT vs. 19.2% T1 in NBRST).

Third, the number of ER+/Basal patients in our study was relatively small and the trial design created some heterogeneity in (neo)adjuvant treatment strategies for these patients. Because of the relatively low number of ER+/Basal patients, it will be difficult to design a prospective randomized clinical trial to define the optimal treatment for this subgroup of patients. Long-term prognosis for these patients is controversial. For borderline ER-positive patients, some studies reported clinical outcomes similar to those of ER negative cancers, and others showing an intermediate prognosis compared to ER-negative and ER-positive tumors.^8^ In MINDACT, the subgroup of ER+/Basal patients (*N* = 99) had a 5-year distant metastasis free survival (DFMS) of 90.2% compared to 95.9% for ER+/Luminal-type patients, although this difference was not statistically significant.^5^ We report a substantially higher rate of pCR to neoadjuvant chemotherapy for this subgroup of ER+HER2−/Basal patients compared to HR + HER2−/Luminal patients (34 vs. 5%), indicating the presence of underlying differences in biology between these subgroups.^7^ The high pCR rate of ER+/HER2−/Basal patients was recently confirmed in the I-SPY 2 trial.^20^ Our preliminary 3-year DMFI analysis also supports that the long-term outcome of these ER+/Basal patients is in-line with clinically Basal-type patients, both in metastasis free survival and in the pattern of early events. Although it is unclear if ER+/Basal tumors are responsive to endocrine therapy, a potential benefit from empirical adjuvant endocrine therapy cannot be excluded in this subgroup of patients. Of significant practical impact, we have identified by genomic profiling a group within clinically ER-positive patients who exhibit a significantly improved response to neoadjuvant chemotherapy (i.e., Basal-type) while also identifying the patients who do likely not benefit from neoadjuvant chemotherapy (i.e., Luminal-type). The issue becomes how the treatment of high-risk Basal patients, irrespective of ER status, would be different from high-risk Luminal patients in both the neoadjuvant and adjuvant setting. In clinical practice, the chemotherapy regimens are more often considered differently based on (molecular) subtype and future studies are needed to specifically address this question. Finally, our study provides a rationale why ER+/Basal breast cancers are transcriptionally ER-negative.

## Methods

### Patient selection NBRST registry trial

The prospective NBRST registry trial (ClinicalTrials.gov identifier NCT01479101) enrolled 1072 breast cancer patients from 64 US institutions between June 2011 and December 2014. NBRST was conducted as an observational, case-only study and exploratory in nature, therefore a sample size calculation was not applied as only descriptive statistics were pre-planned. Patients received either neoadjuvant chemotherapy (NCT) or neoadjuvant hormonal therapy (NHT) after successful BluePrint molecular subtyping and MammaPrint genomic risk stratification (Agendia Inc). Selection of neoadjuvant and adjuvant treatment regimen was at the discretion of physicians and no specific recommendations were given. The trial was approved by Institutional Review Boards in all participating institutions. Clinical data collection and consent for use of tissue for future scientific research was obtained by written informed consent.

### Clinical and molecular subtyping

Hormone receptor status was locally assessed on pretreatment core biopsies using immunohistochemistry for estrogen receptor (ER) and progesterone receptor (PR). In accordance with the joint ASCO/ACP guideline, both ER and PR were considered positive if these was ≥1% tumor cells with positive nuclear staining.^1^ HER2 status was locally assessed by immunohistochemistry and/or fluorescence in situ hybridization (FISH). HER2 status was regarded as positive if there was 3+ staining and/or FISH positivity.

Molecular subtyping was performed using the 80-gene molecular signature BluePrint (Agendia Inc.) in a centralized laboratory on RNA isolated from pretreatment fresh frozen or formalin-fixed core needle biopsies. Genomic risk assessment was performed on the same RNA sample using the 70-gene molecular signature MammaPrint (Agendia Inc), that stratified patients at low or high risk for disease recurrence. Both assays are microarray based and specific procedures has been described before.^18,25^

### Variant specific quantitative real-time PCR (qPCR)

ERα variant specific primer pairs were designed in which the reverse primer was located across the splice site (ER∆3 and ER∆7) or in a variant specific exon (ERα-36). For total ERα mRNA, an intron-spanning primer pair was designed in which the forward primer was located in exon 1 and the reverse primer in exon 2. Primer sequences are shown in Supplementary Table 1.^16^ Specificity of the primer pairs was tested using cDNA from MCF7 breast cancer cells overexpressing either wild-type or variant ERα.

cDNA was synthesized for all samples in two independent reactions each using 500 ng RNA (fresh frozen samples), 800 ng RNA (FFPE samples) or 125 ng RNA (fresh frozen normal breast controls) using SuperScript IV reverse transcriptase (ThermoFisher) with random hexamers following manufacturer’s protocol (50 min reaction time). qPCR reactions were performed in technical triplicates using PowerUp SYBR Green (ThermoFisher) master mix in a 15 ul reaction with 0.8 ul 10 μM primermix and 5 μl diluted cDNA. qPCR reactions were performed using the 7500 Fast system (Applied Biosystems) and analyzed using the 7500 Software v2.3 (Applied Biosystems). Expression levels were calibrated and quantified using a reference standard dilution curve (7-fold log_10_ concentration range) from a synthetic qPCR template containing the sequence template for all the reactions (gBlock, Integrated DNA Technologies). Two internal reference genes were used for normalization (GAPDH and Actin-B). The relative expression of ERα variants was calculated by dividing the variant mRNA expression by the total ERα mRNA expression.

### Sample selection ERα variant analysis

In the NBRST study, RNA was available from 84 out of 91 ER-positive Basal-type tumors (Fresh Frozen *N* = 18, FFPE *N* = 66) for quantification of total ERα mRNA and ERα variant mRNA by qPCR. All 84 cases were classified by MammaPrint as high-risk. RNA from 3 cases was of too low quality for successful ER variant analysis. For comparison, we selected from the NBRST study 48 ER-positive, MammaPrint high-risk Luminal-type (i.e., Luminal B) tumors with a broad range of ER-positivity by IHC (range 1–100%) for which RNA was available (Fresh Frozen sampes *N* = 8, FFPE samples *N* = 40). As a second control group, we used an external set of 8 fresh frozen RNAs isolated from histologically verified normal breast tissue from 7 patients (range 40–72 years) with concurrent breast cancer (Erasmus MC, Rotterdam, The Netherlands).

### Treatment response and outcome

Response to treatment was collected in institutionally approved case report forms (CRF) following definitive surgery after completing neoadjuvant therapy, and then again 2–3 years and 5 years after surgery. Pathological complete response (pCR) was used as the primary endpoint to assess response to the neoadjuvant treatment regimen, defined as the absence of invasive carcinoma in both the breast and axilla at microscopic examination of the resection specimen, regardless of the presence of carcinoma in situ (ypT0/TisN0).^26^ Distant metastasis free interval (DMFI) was used as the primary endpoint for long-term follow-up, defined as time from diagnosis to distant metastasis or time of breast cancer-related or unknown cause of death.^27^

### Statistical analysis

Data analysis and statistics were performed using Prism 7 software. Differences between groups were compared using the Mann–Whitney test (2-sided *p*-value). Kaplan–Meier survival analysis and log-rank statistics were performed in R version 3.4.0.

### Reporting summary

Further information on experimental design is available in the Nature Research Reporting Summary linked to this article.

## Supplementary information

Supplementary Information

Reporting Summary

## Data Availability

The clinical and expression analysis data files generated and analyzed during this study are available in the following data record: 10.6084/m9.figshare.7855742.^16^ The data record also includes a description of data files not available in the figshare repository, along with relevant data access requirements. As outlined in the data record, certain data files supporting this analysis are available on request only; raw array data have not been made publicly available as part of a collaboration agreement with the diagnostic company Agendia Inc.

## Supplementary information

**Supplementary information** accompanies the paper on the *npj Breast Cancer* website (10.1038/s41523-019-0109-7).
