# Supplementary material for: Estrogen receptor variants in ER-positive basal-type breast cancers responding to therapy like ER-negative breast cancers
Source: NPJ Breast Cancer. 2019 Apr 18;5:15. doi: 10.1038/s41523-019-0109-7 (PMC6472385; doi:10.1038/s41523-019-0109-7)
Supplement: Supplementary file 2 — Reporting Summary [file 41523_2019_109_MOESM2_ESM.pdf]

**Supplementary Information**

**Supplementary Table 1……………………………………………………………..Page 2**

**Supplementary Figure 1……………………………………………………………Page 3**

**Supplementary Figure 2……………………………………………………………Page 4**

**Supplementary Figure 3……………………………………………………………Page 5**

**Supplementary Table 1.** Primer sequences used for ER variant analysis by qPCR

| **Gene/variant** | **Primersequence (5'->3')** |
|---|---|
| **GAPDH**<br>108bp | Forward:<br>AAGGTGAAGGTCGGAGTCAA |
| | Reverse:<br>AATGAAGGGGTCATTGATGG |
| **ACTB**<br>93bp | Forward:<br>GCACAGAGCCTCGCCTT |
| | Reverse:<br>GTTGTCGACGACGAGCG |
| **Total ER-alpha66**<br>101bp | Forward (exon 1):<br>CAGGTGCCCTACTACCTGGAGAA |
| | Reverse (exon 2):<br>CCCTGGCGTCGATTATCTGAATTTGG |
| **ER∆3**<br>107bp | Forward (exon 2):<br>CTGTGCAGTGTGCAATGACT |
| | Reverse (splice site exon 2 (10nt) and exon 4 (15nt)):<br>GGTCTTTTCGTATCCCTTGAATACT |
| **ER∆7**<br>101bp | Forward (exon 6):<br>TGCTGGCTACATCATCTCGGTT |
| | Reverse (splice site exon 6 (12nt) and exon 8 (14nt)):<br>CCATGCCTTTGTTACAGAATTAAGCA |
| **ER-alpha36**<br>112bp | Forward (exon 6):<br>ACATGCTGCTGGCTACATCA |
| | Reverse (splice site exon 6 (6nt) and variant specific exon (19nt)):<br>GCTTCTACATGTGAGATACCAGAAT |

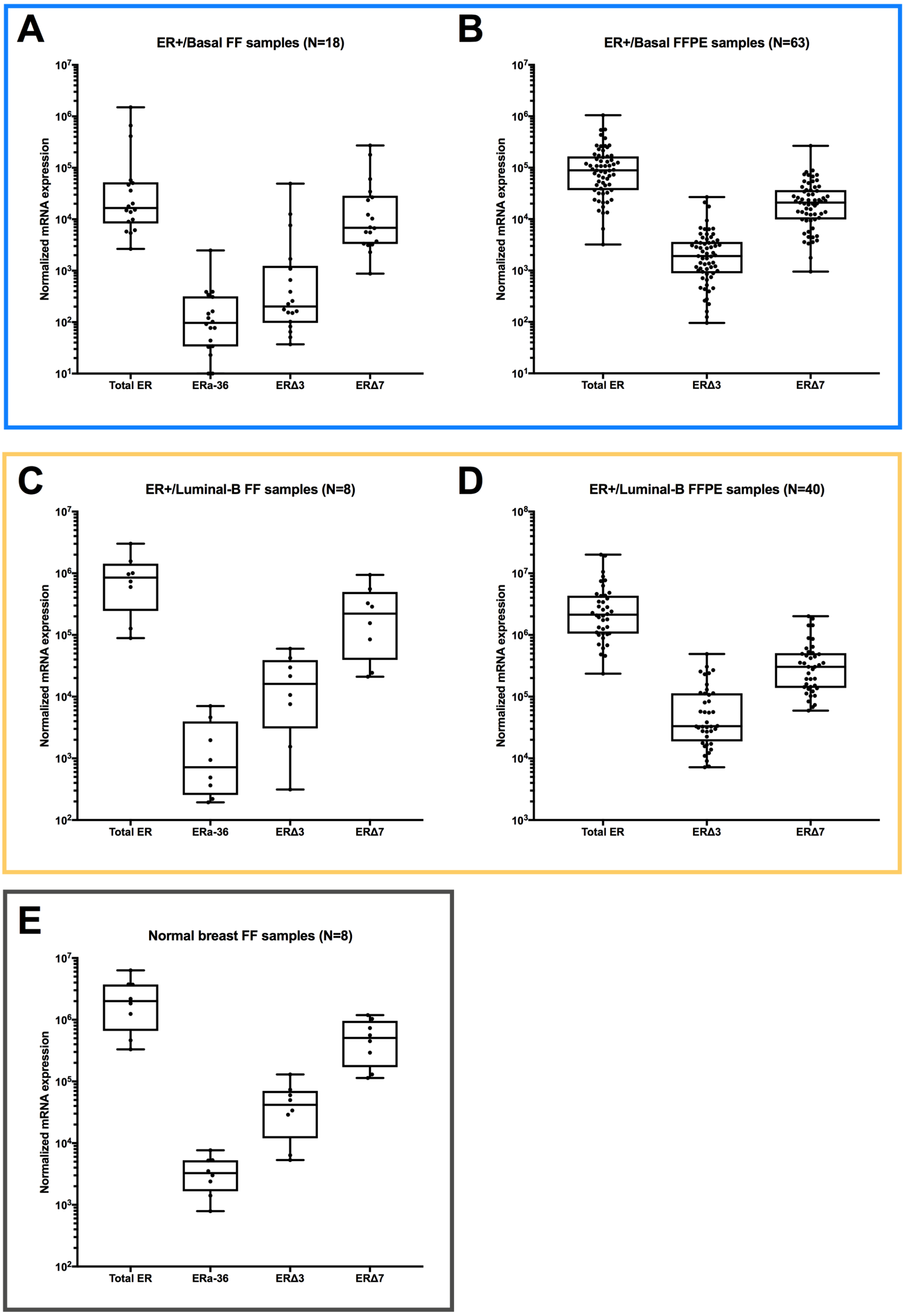


**Supplementary Figure 1. Normalized total ERalpha and ER variants mRNA expression**

Box plots showing normalized total ERalpha and ER variants mRNA expression for ER+/Basal tumors (A and B, blue box), ER+/Luminal B tumors (C and D, orange box) and normal breast tissues (E, grey box). Normalized ERα-36 mRNA expression is only shown for fresh frozen (FF) samples (A, C and E), since the expression was below the detection limit in formalin-fixed paraffin embedded (FFPE) samples (B and D). Central line in boxes represent the median value, boundaries of boxes represent the interquartile range and ends of whiskers represent the minimum and maximum values.

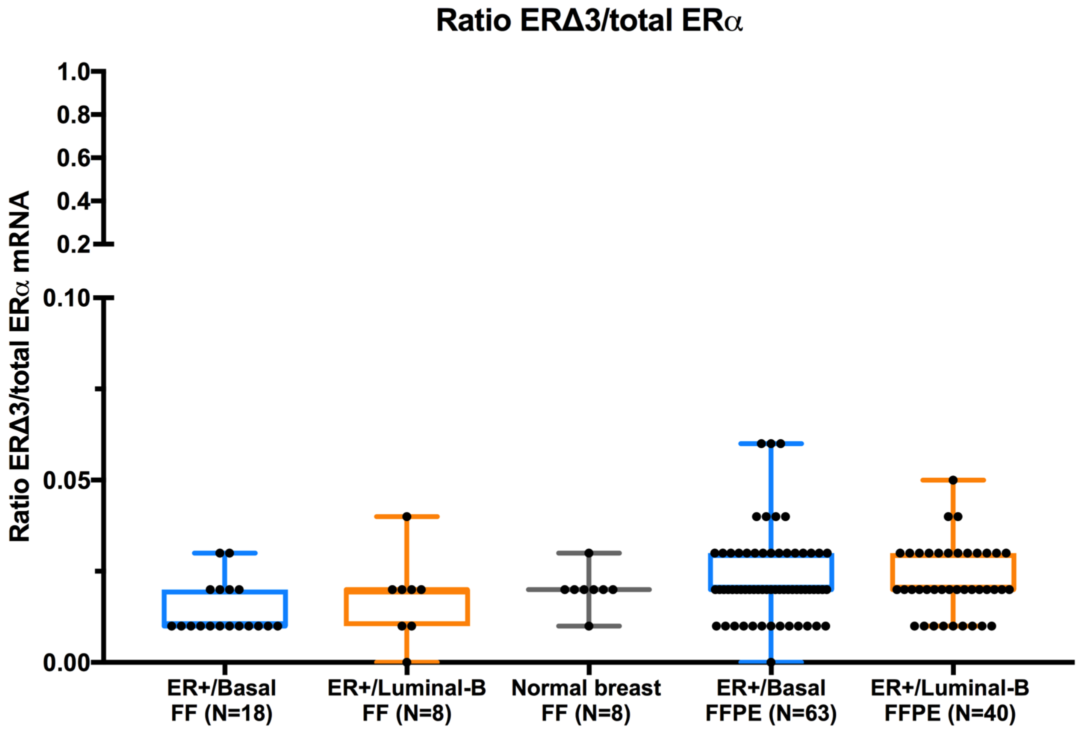


**Supplementary Figure 2. Ratio of ERΔ3 mRNA expression and total ERalpha mRNA expression**

Box plots showing ratio of ERΔ3 mRNA expression and total ERalpha mRNA expression for ER+/Basal tumors (blue boxes), ER+/Luminal B tumors (orange boxes) and normal breast tissues (grey box). Ratio's for fresh frozen (FF) samples and formalin-fixed paraffin embedded (FFPE) samples are depicted in separate boxplots. Central line in boxes represent the median value, boundaries of boxes represent the interquartile range and ends of whiskers represent the minimum and maximum values.

A

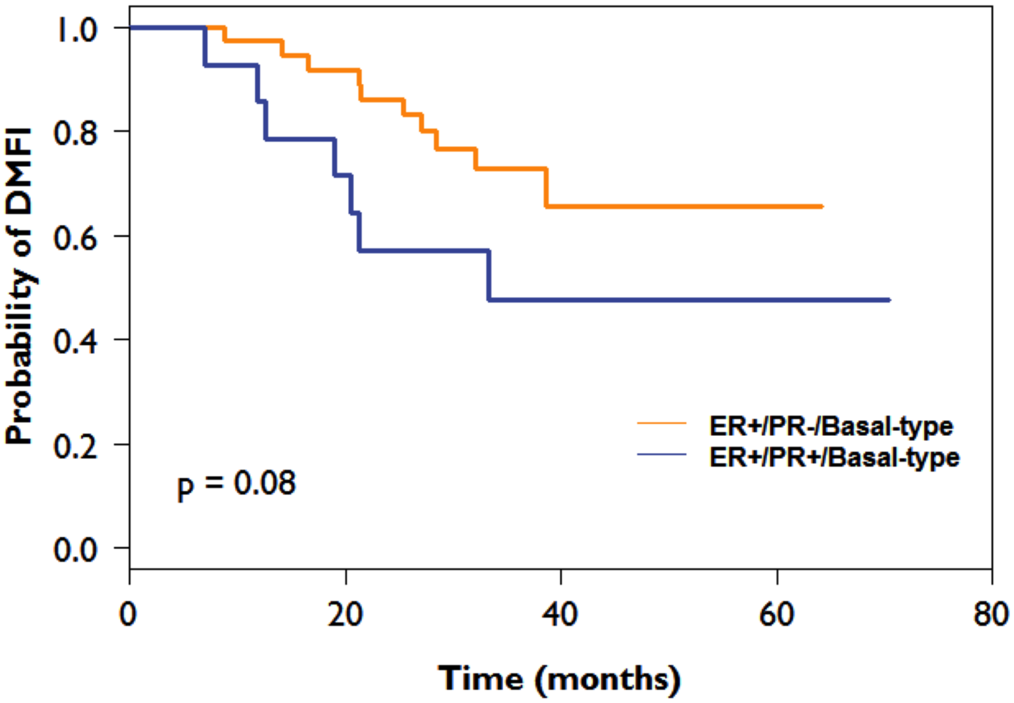


B

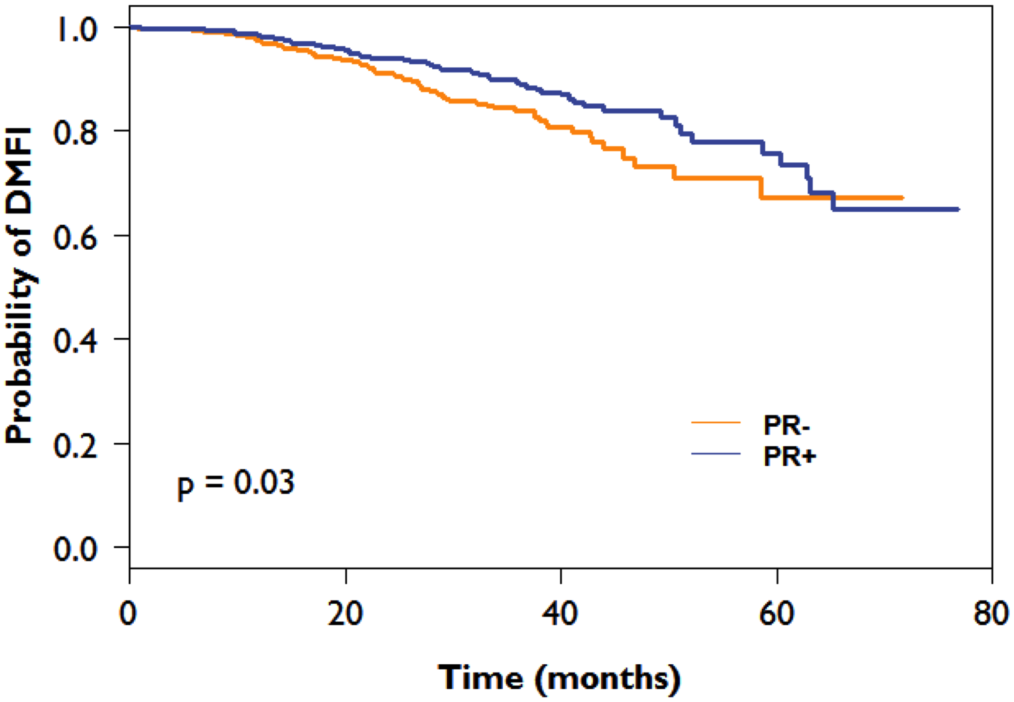


**Supplementary Figure 3. Kaplan Meier curves showing distant metastasis free interval (DMFI)**
**A,** DMFI for ER+/Basal patients (N=70) stratified by PR status. **B,** DMFI for all HER2-negative patients in NBRST study with follow-up (N=538) stratified by PR status. P-values are obtained using the log-rank test.
